# Induction of LEF1 by MYC activates the WNT pathway and maintains cell proliferation

**DOI:** 10.1186/s12964-019-0444-1

**Published:** 2019-10-17

**Authors:** Yi-Heng Hao, M. Carmen Lafita-Navarro, Lauren Zacharias, Nofit Borenstein-Auerbach, Min Kim, Spencer Barnes, Jiwoong Kim, Jerry Shay, Ralph J. DeBerardinis, Maralice Conacci-Sorrell

**Affiliations:** 1 Department of Cell Biology, UT Southwestern Medical Center, Dallas, TX 75390 USA; 2 Howard Hughes Medical Institute and Children’s Research Institute, UT Southwestern Medical Center, Dallas, TX 75390 USA; 3 Lyda Hill Department of Bioinformatics, UT Southwestern Medical Center, Dallas, TX 75390 USA; 4 Harold C. Simmons Comprehensive Cancer Center, UT Southwestern Medical Center, 76092, Dallas, TX USA; 5 Hamon Center for Regenerative Science and Medicine, UT Southwestern Medical Center, Dallas, 76092 TX USA

**Keywords:** MYC, WNT/β-catenin pathway, LEF1, Colon cancer, Tumorigenesis, Metabolism, PPARδ, ACAD9, Proliferation

## Abstract

**Background:**

While regulated WNT activity is required for normal development and stem cell maintenance, mutations that lead to constitutive activation of the WNT pathway cause cellular transformation and drive colorectal cancer. Activation of the WNT pathway ultimately leads to the nuclear translocation of β-catenin which, in complex with TCF/LEF factors, promotes the transcription of genes necessary for growth. The proto-oncogene MYC is one of the most critical genes activated downstream the WNT pathway in colon cancer. Here, we investigate the converse regulation of the WNT pathway by MYC.

**Methods:**

We performed RNA-seq analyses to identify genes regulated in cells expressing MYC. We validated the regulation of genes in the WNT pathway including LEF1 by MYC using RT-qPCR, Western blotting, and ChIP-seq. We investigated the importance of LEF1 for the viability of MYC-expressing cells in in fibroblasts, epithelial cells, and colon cells. Bioinformatic analyses were utilized to define the expression of MYC-regulated genes in human colon cancer and metabolomics analyses were used to identify pathways regulated by LEF1 in MYC expressing cells.

**Results:**

MYC regulates the levels of numerous WNT-related genes, including the β-catenin co-transcription factor LEF1. MYC activates the transcription of LEF1 and is required for LEF1 expression in colon cancer cells and in primary colonic cells transformed by APC loss of function, a common mutation in colon cancer patients. LEF1 caused the retention of β-catenin in the nucleus, leading to the activation of the WNT pathway in MYC-expressing cells. Consequently, MYC-expressing cells were sensitive to LEF1 inhibition. Moreover, we describe two examples of genes induced in MYC-expressing cells that require LEF1 activity: the peroxisome proliferator activated receptor delta (PPARδ) and the Acyl CoA dehydrogenase 9 (ACAD9).

**Conclusions:**

We demonstrated that MYC is a transcriptional regulator of LEF1 in colonic cells. Our work proposes a novel pathway by which MYC regulates proliferation through activating LEF1 expression which in turn activates the WNT pathway.

**Graphical Abstract:**

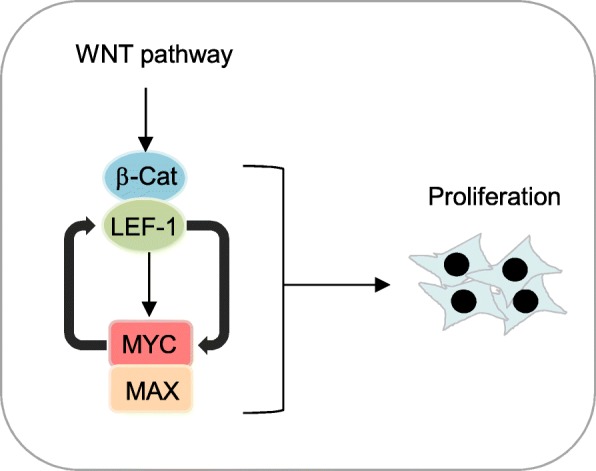

## Background

Colorectal cancer results from the accumulation of mutations in specific genes, including adenomatous polyposis coli (*APC*), *K-RAS*, and *P53* [1]. These mutations drive the transition from normal colonic epithelia to dysplastic adenoma and colorectal carcinoma [2]. Mutations in the *APC* gene are responsible for familial adenomatous polyposis (FAPC) and are also involved in the initiation of the majority of sporadic colorectal cancers [3]. The primary tumor suppressive role of APC is to negatively regulate the WNT signaling pathway via its role as a scaffold for the β-catenin destruction complex [4].

In normal cells, activation of the canonical WNT pathway occurs when secreted WNT ligands bind to the Frizzled and LRP5/6 membrane receptors, leading to the activation of a signaling cascade that promotes the nuclear translocation of β-catenin [5, 6]. In the nucleus, β-catenin interacts with transcription factors from the T-cell factor/Lymphoid enhancer factor (TCF/LEF) family and drives the expression of genes involved in cell proliferation, migration, and embryonic development [7]. TCF/LEF transcription factors are broadly expressed during embryonic development, during which they mediate physiological WNT signaling [8]. TCF/LEF proteins also mediate WNT signaling in adult tissues, especially in tissues derived from stem cell populations [8].

Regulated degradation of β-catenin limits WNT activity and suppresses cellular transformation. Cytosolic β-catenin is phosphorylated by a complex containing the scaffold molecules AXIN and APC together with the kinases glycogen synthase kinase 3β (GSK3β) and casein kinase 1 (CK1) [9]. The consecutive phosphorylation by CK1 and GSK3β targets β-catenin for proteolytic degradation by the proteasome [10]. Mutations in APC or AXIN that impair β-catenin degradation upregulate WNT signaling, leading to hyperproliferation and facilitating colon cancer development [7].

In addition to the activation of the WNT pathway via mutations in APC, intracellular TCF/LEF composition and localization also contribute to WNT oncogenic activity in colonic cells [8]. In normal colon, the family members TCF1 and TCF4 are expressed, while LEF1 and TCF3 loci are silent [11]. However, the levels of LEF1 mRNA and protein are significantly greater in human colon cancer tissues, and LEF1 knockdown in xenograft models suppresses tumor formation and growth [12]. In complex with β-catenin, TCF/LEF transcription factors activate the transcription of oncogenes such as cyclin D1 and MYC, which mediate cellular transformation [13–15].

The transcription factor MYC is a universal oncogene and a major driver of metabolic reprograming in tumors [16]. MYC induces the transcription of genes necessary for the uptake and synthesis of fatty acids, amino acids, and nucleotides to provide building blocks for continuously proliferating cells [17, 18]. Because MYC plays a widespread role in regulating metabolism and the cell cycle, aberrant activation of MYC family member (MYC, MYCN, MYCL) by DNA amplification, transcriptional upregulation, or protein stabilization contributes to tumorigenesis [17, 19–23]. For over two decades MYC has been studied as the most critical pro-proliferative target gene of the WNT pathway in colon cancer [14, 24]. Yet, the impact of MYC on the WNT pathway is not fully known. Here we report the discovery that MYC regulates the expression of genes in the WNT pathway, including *LEF1*, leading to the nuclear localization of β-catenin and the activation of the WNT pathway in proliferating cells.

## Methods

### Cell culture and proliferation

Rat1 fibroblasts, human epithelial cell line ARPE-19, human colon cancer cell lines DLD1, RKO and HCT116, human pancreatic cancer cell line Colo357, and human liver cancer cell line Huh7 were cultured in DMEM with 5% FBS, 100 U/mL pen/strep. Human colonic epithelial cells (HCEC) with truncated APC, KRas V12 mutation, or P53 knockdown were cultured as described before [25]. Colon cancer-related alterations in cell lines used in in this study as described in Additional file 4: Figure S4B. Cell lines, apart from HCEC, were obtained from ATCC and are mycoplasma free. Cell proliferation was measured by plating either 5,000 ARPE-19 or cancer cells, or 20,000 HCEC cells or Rat1 fibroblasts in 12-well plates, transfecting with siRNA or adding WNT inhibitors, and staining with crystal violet 1–7 days after plating. Cell densities were quantified using ImageJ [26]. For time course study, cells were plated on the same day and inhibitors or DMSO were added on indicated days before the cells were fixed and stained with crystal violet. All experiments were performed at a minimum of three times and each experiment contained tree technical replicates. Statistical significance was analyzed by t-test and statistical significance was established by a *p* = 0.05 for every experiment.

### TOPFlash luciferase assays

For the LEF/TCF promoter activity assays, 1.5 × 10^5^ control DLD1 cells and DLD1 cells with MYC overexpression or knockdown seeded in triplicate in 6-well plates were transfected with the M50 Super 8X TOPFlash luciferase reporter plasmid (Addgene) with seven TCF/LEF binding [27] or control M51 Super 8X FOPFlash plasmids (Addgene) (with seven copies of mutated TCF/LEF consensus sequence). 24 h after transfection, cells were treated with DMSO or 20 μM ICG-001 overnight, before being lysed with Glo Lysis Buffer (Promega). Luciferase activity was measured with ONE-Glo™ Luciferase Assay System (Promega) and normalized with FOPFlash samples. All experiments were performed at least three times and each experiment contained three technical replicates. Statistical analyses were performed by t-test and statistical significance was established by a *p* = 0.05 for every experiment.

### Metabolomics analyses

Qualitative assessment of global metabolites by mass spectrometry (metabolomics) were performed by the metabolomics facility at the Children’s Research Institute (UT Southwestern Medical Center, UTSW) as previously described [28, 29]. Metabolomics were performed 48 h after LEF1 siRNA transfection, when LEF1 silencing was efficient, but no cell death has occurred. The experiment was performed in triplicate, and the PCA plot shows that replicates cluster closely (Additional file 8: Figure S8C). Using a cut-off of 30% changes and VIP score above 1.

### Western blotting and nuclear and cytoplasmic fractionation

To knockdown target genes, 50 nM of siRNA was reverse transfected to 2.5 × 10^5^ cells in 6-well plates with Lipofectamine RNAiMAX reagent. Total protein lysates were extracted with NP40 Lysis buffer (50 mM Tris-HCl pH 7.7, 150 mM NaCl, 0.5% NP-40) with DTT and protease inhibitors. Cells transfected with siRNA or treated with 20 μg/mL Leptomycin B (LMB) for 3 h were fractionated into nuclear and cytoplasmic fractions as described previously (Conacci-Sorrell et al. 2010) and subjected to Western blotting. Antibodies used for Western blotting were: anti-MYC (Abcam AB32072), anti-LEF1 (Cell Signaling 2230), anti-LRP6 (Cell Signaling 2560), anti-TCF4 (Cell Signaling 2569), anti-DVL2 (Cell Signaling 3224), anti-LIF (Abcam AB135629), anti-β-catenin (Abcam AB32572), anti-Axin2 (Abcam AB109307), anti-MAX (Sant Cruz sc-197), anti-α-Tubulin (Sigma T6199), anti-β-actin (Cell Signaling 8457), anti-mSin3A (Santa Cruz sc-994), anti-Histone H3 (Cell Signaling 4499), anti-Cleaved Caspase-3 (Cell Signaling 9664), anti-Cyclin A1 (Novus Biologicals, MAB7046), anti-PPARδ (ThermoFisher PA1-823A), anti-ACAD9 (Cell Signaling 9796S), and anti-ACADS (ThermoFisher PA5–54580).

Human colon tumor tissues and their surrounding benign tissues obtained from the Tissue Management Shared Resource at Harold C. Simmons Comprehensive Cancer Center (UTSW) were ground and lysed with NP40 Lysis buffer, and the total lysates were subjected to Western blotting.

### RNA-seq and RT-qPCR

Rat1 fibroblasts with *myc*−/− expressing vector or reconstituted with human MYC were collected and sent to GENEWIZ for RNA extraction and sequencing and results was analyzed as previously published [30]. Raw data is accessible at NCBI GEO (GSE135061). RNA-seq assessment of the raw sequencing reads was done using the NGS-QC-Toolkit [31]. The reads were aligned to the genome RGSC 6.0/rn6 using HISAT2 (v 2.1.0) aligner. A minimum read count filter of 10 total reads was applied to remove low-expressed genes. Filtered reads were then normalized using DESeq2 [32]. DeSeq2 employs a negative binomial distribution to estimate data variability and uses an error model for a more robust statistical test for significance. An FDR cutoff of < 5% was used to select significantly altered genes between experiment conditions. Primers used for qPCR are listed in Additional file 9: Table S1. All experiments were performed at least three times and each experiment contained three technical replicates. Statistical analyses were performed by t-test and statistical significance was established by a *p* = 0.05 for every experiment.

### Chromatin immunoprecipitations

ChIP was performed with anti-MYC antibody (Y69) as described previously (Lafita-Navarro et al. 2018). Briefly, 60 × 10^6^ DLD1 cells were fractionated and purified nuclei were sonicated to shear chromatin in sizes of 200 to 600 bp. Purified chromatin fragments were incubated with the anti-MYC antibody (Y69) overnight. DNA fragments binding to the antibody were pulled down with Protein G magnetic beads and purified for RT-PCR assays. All experiments were performed at least three times and each experiment contained three technical replicates. Statistical analyses were performed by t-test and statistical significance was established by a *p* = 0.05 for every experiment.

### Inducible MYC expression and protein synthesis inhibition

Rat fibroblast cell line with inducible MYC expression (Tet-On) was cultured with or without the presence of 1 μg/ml doxycycline (Dox) for 48 h. Protein synthesis was inhibited by incubating with 25 μg/ml cycloheximide (CHX) for three hours before cells were harvested.

### Bioinformatics TCGA analyses

PANCAN normalized gene expression data for various cancer types were downloaded from the TCGA Research Network. Heatmaps were generated without additional modification to the downloaded data using R statistical analysis tools. Tumor samples were compared to the corresponding normal tissue sample from each patient. Significance testing between groups was performed using nonparametric Kruskal-Wallis test. Kaplan–Meier survival curves were generated for comparison between patients that had the 30% highest and 30% lowest levels of expression of MYC-driven WNT target genes. Significance was defined as*p*-value < 0.05 using ONCLnc (http://www.oncolnc.org/).

## Results

### MYC regulates the expression of genes in the WNT pathway

**Fig. 1:**
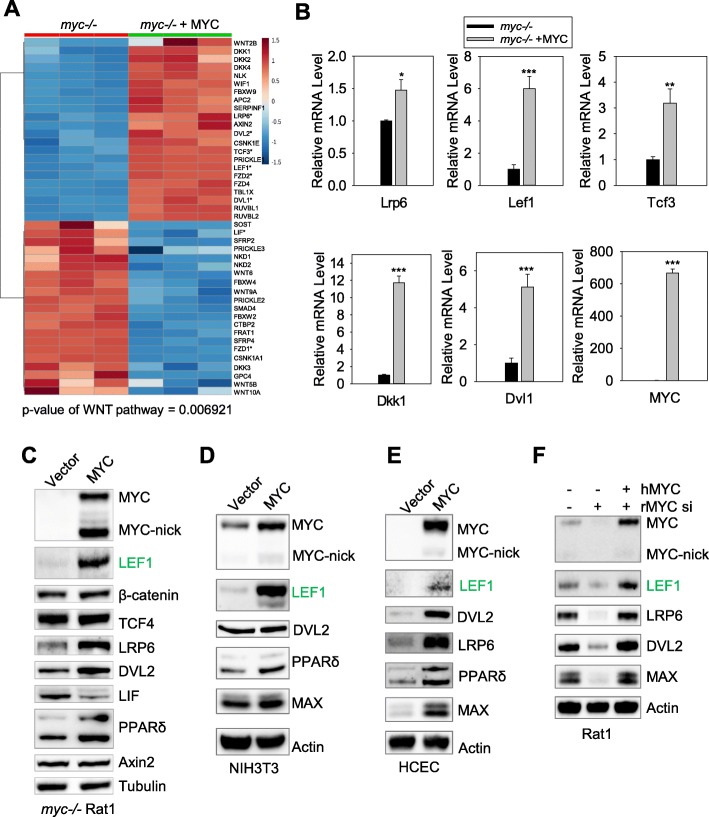
MYC regulates the expression of components of the WNT pathway. **a** Rat1 *myc−/−* fibroblasts expressing either vector or human MYC were collected and subjected for RNA-seq analysis. The heatmap was generated based on relative expression of each sample for the members of the WNT pathway regulated by MYC with cutoff of Log_2_FC = 0.585. Asterisks denote genes verified by using RT-qPCR or Western blotting. **b** RT-qPCR for the indicated genes in Rat1 fibroblasts *myc*−/− or Rat1 fibroblasts *myc*−/− reconstituted with human MYC. Expression levels of each gene was normalized to the levels of 18S, and the expression levels of each genes in *myc*−/− samples was set to 1. **c**-**e** Rat1 fibroblasts (**c**), NIH3T3 cells (**d**), and human colonic epithelial cells (HCEC, **e**) were extracted with NP40 lysing buffer, and the total cell lysates were subjected to Western blotting with the indicated antibodies. **f** Parental Rat1 fibroblasts with or without human MYC (hMYC) overexpression were transfected with control or MYC siRNAs for 3 days before protein extraction and Western blotting. * *p* < 0.05, ** *p* < 0.01, *** *p* < 0.001

To identify transcriptional networks downstream of MYC, we performed an RNA sequencing (RNA-seq) experiment comparing *myc*−/− Rat1 fibroblasts with and without reconstitution using human MYC. This approach revealed novel transcription factors that mediate MYC functions [30]. With a log2FC of 0.585 and adjusted p-value of 0.01 as cutoff, we demonstrate that expression levels of over 5000 genes are altered by MYC [30]. KEGG pathway enrichments analysis identified the WNT signaling pathway as one of the pathways significantly altered by MYC with a *p* = 0.006921 (Fig. 1a, Additional file 1: Figure S1A). Among the genes activated by MYC were *WNT2B*; the membrane WNT receptors, *FZD2* and *LRP6*; and the transcriptional co-factor of β-catenin *LEF1* (Fig. 1a). The overall signature of genes regulated by MYC suggests that MYC activates the WNT signaling pathway (Additional file 1: Figure S1A).

Because MYC transcription is induced by the binding of β-catenin complexed with TCF/LEF to the MYC promoter [14], we used cells expressing MYC under the control of an exogenous promoter, the retroviral vector pBabe. This allowed us to study the effects of MYC on WNT pathway genes independently from the activation of MYC by β-catenin-TCF/LEF. Using *myc−/−* Rat1 fibroblasts expressing empty vector or MYC, we validated the RNA-seq results by performing RT-qPCR and Western blotting for randomly selected genes (Fig. 1 b, c, Additional file 1: Figure S1B). As expected, we found that FZD2, LRP6, LEF1, and TCF3 were induced by MYC, while the levels of FZD1 and LIF were repressed in MYC-expressing fibroblasts, thus confirming our RNA-seq results (Fig. 1 b, c, Additional file 1: Figure S1A-B).

MYC overexpression in a normal human retinal epithelial cell line (ARPE-19) also induced the expression of WNT-related genes, including LRP6, LEF1 and TCF3, as confirmed by RT-qPCR and Western blotting (Additional file 1: Figure S1C, E). Expression of MYC in the mouse fibroblast line NIH3T3 induced LEF1 expression but not DVL2 (Fig. 1d). LRP6 was undetectable. Expression of MYC in normal colonic epithelial cells (HCEC) led to the expression of LEF1, DVL2 and LRP6 (Fig. 1e). Silencing or knocking out endogenous MYC in wild-type (WT) Rat1 fibroblasts led to downregulation in LEF1, LRP6 and DVL2, which was rescued by the expression of human MYC that was not targeted by the rat MYC siRNA (Fig. 1f and Additional file 1: Figure S1D).

We previously reported that MYC induces the expression of the transcription factor aryl hydrocarbon receptor (AHR), which mediates the transcription of approximately 300 genes in MYC-expressing cells [30]. In the current study, RNA-seq analysis showed that genes related to the WNT pathway such as LEF1 were regulated by MYC independently of AHR (Additional file 1: Figure S1F). The MYC co-transcription factor MAX, which is also induced by MYC in our RNA-seq experiments [30], was used as a positive control on Western blots (Fig. 1 d-f). Our results indicate that the induction of WNT-related genes by MYC is likely to be a general mechanism to control WNT activity in normal cells. The WNT-related gene LEF1 was the most robustly induced by MYC in all cell lines evaluated (Fig. 1, Additional file 1: Figure S1) at both mRNA and protein levels. Therefore, we have focused our study on the regulation of LEF1 by MYC and on the molecular roles played by LEF1 in MYC-dependent cells.

### LEF1 is regulated by MYC in colon cancer cells

**Fig. 2:**
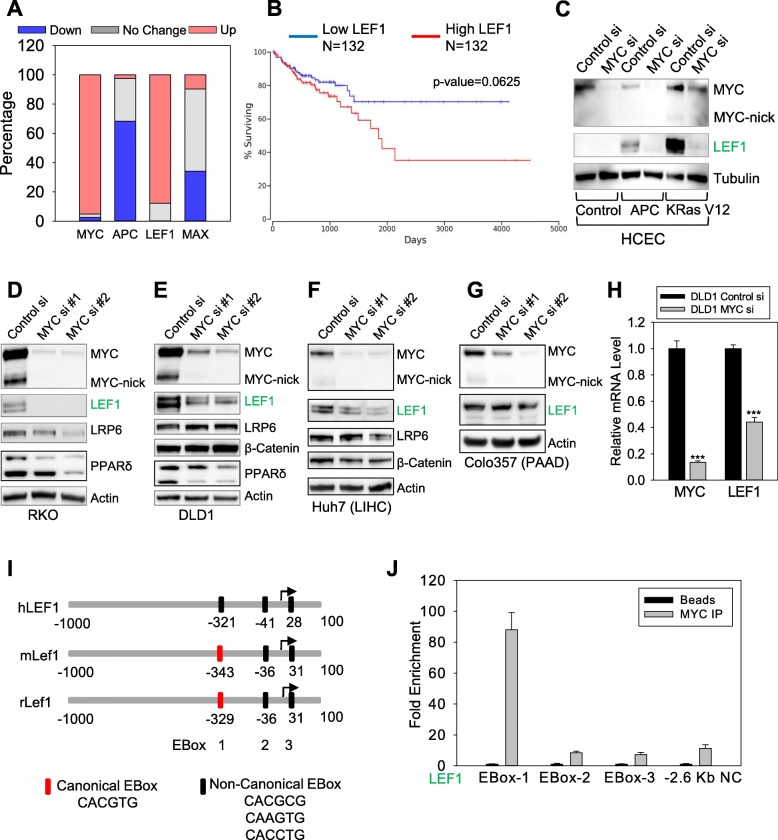
MYC-regulated genes in the WNT pathway are upregulated in cancer. **a** Comparison of the levels of the indicated genes in cancer tissues and adjacent normal tissues from TCGA database. Blue: downregulated (< 0.7), grey: No change (0.7–1.3), and red: upregulated (> 1.3), *N* = 41 pairs. **b** Kaplan–Meier curve comparing survival of patients with colon adenocarcinoma (COAD) and high or low levels of LEF1. Comparison between 30% highest and 30% lowest expression were generated using ONCLnc (http://www.oncolnc.org/). **c** HCEC expressing control vector, truncated APC, or constitutively activated KRas were transfected with control or MYC siRNAs, and total proteins were extracted with NP40 lysing buffer for Western blotting with the indicated antibodies. **d**-**g** RKO (**d**), DLD1 (**e**), Huh7 (**f**), and Colo357 cells (**g**) were transfected with control or MYC siRNAs, and total proteins were extracted with NP40 lysing buffer for Western blotting with the indicated antibodies. **h** DLD1 cells were transfected with either control or MYC siRNAs, and the relative RNA levels were measured by using RT-qPCR. Expression levels of each gene was normalized to the levels of 18S, and expression levels of each gene in the control siRNA samples was set to 1. **i** Identification of the MYC-specific binding motif E-Box in the promoter regions of human, mouse, and rat LEF1. Canonical EBox (CACGTG), non-canonical EBox (CACGCG, CAAGTG, or CACCTG). **j** ChIP for MYC (Y69 antibody) on the promoter regions of LEF1 and surrounding upstream region that do not contain Eboxes (− 2.6-kb) in DLD1 cells. Fold enrichment for MYC immunoprecipitation in comparison with control was determined by qPCR. * *p* < 0.05, ** *p* < 0.01, *** *p* < 0.001

Because the activity of both MYC and the WNT pathway are profoundly involved in colon cancer [8, 15], we examined the 41 pairs of normal/tumor colonic tissues deposited in the TCGA database for the expression of LEF1 and other genes upregulated by MYC in our RNA-seq dataset (Fig. 1a). MYC mRNA levels were higher in 39 of the 41 tumor samples than the normal mucosa of the same patients, while *APC* mRNA levels were lower in the majority of cancer samples (Fig. 2a). As opposed to MYC, MAX levels were not consistently altered in colon cancer patient samples. MAX levels are unaffected in over 50% of the samples, reduced in 34% and increased in about 10%. Importantly, most of WNT pathway genes induced by MYC, including *DVL1, LEF1, RUVBL1*, and *RUVBL2*, were also upregulated in tumor tissues when compared with the normal tissues from the same patients (Fig. 2a and Additional file 2: Figure S2A), suggesting that these genes may play a major role in colon cancer cells.

To determine whether MYC-regulated genes affect prognosis of colon cancer patients, we generated Kaplan-Meier survival curves comparing patients with colon adenocarcinoma (COAD) expressing high (top 30%) or low (bottom 30%) mRNA levels for the signature of MYC-dependent WNT-related genes. Using this approach, we found that only high levels of the MYC target genes *LEF1* (*p* = 0.0625) (Fig. 2b) and *DVL2* (Additional file 2: Figure S2B) were associated with poorer prognosis. We also examined the expression levels of *LEF1* and other MYC-driven WNT-related genes in all solid tumors available in the TGCA database. Strikingly, we found that the levels of *LEF1, RUVBL1* and *RUVBL2* were elevated in the majority of tumors (Additional file 3: Figure S3A). *LEF1* levels correlated with poor prognosis of various solid tumors including lung and kidney cancers (Additional file 3: Figure S3B).

Examining a previously established collection of the normal colonic cells (HCEC) expressing the known oncogenic drivers of colon cancer such as truncated APC and mutant KRAS (Additional file 4: Figure S4A) [25], we demonstrated that LEF1 was upregulated upon the expression of truncated APC and activated KRAS. Knocking down MYC reduced LEF1 protein levels in both control HCEC and HCEC expressing oncogenes, suggesting that LEF1 is a target of MYC in physiological conditions and in transformed cells (Fig. 2c).

Knocking down MYC reduced LEF1 protein in colon cancer cells such as RKO and DLD1 (Fig. 2 d, e). To determine whether MYC-dependent expression of LEF1 can be generalized to cancer cells derived from tissues other than colon, we silenced MYC in the liver cancer line Huh7 and the pancreatic cancer cell line Colo357 and found that expression of LEF1 was reduced in Huh7 cells, but not as dramatically in Colo357 cells (Fig. 2 f, g).

### MYC directly binds to LEF1 promoter and regulates its transcription

MYC knockdown reduced LEF1 mRNA expression in DLD1 cells (Fig. 2h), thus indicating that LEF1 is transcriptionally regulated by MYC in colon cancer. Similar to LEF1, other WNT-related genes found in our RNA-seq to be regulated by MYC such as FOXQ1 and LRP6, were also transcriptionally repressed upon MYC knockdown in colon cancer cells (Additional file 4: Figure S4C). In a cell line expressing Tet-inducible MYC, we found that LEF1 mRNA is upregulated upon MYC induction (Additional file 4: Figure S4E). These results suggest that MYC expression controls the transcription of genes in the WNT pathway and maintains WNT activity.

MYC-induced genes often contain E-Boxes in their regulatory regions where MYC, in complex with its heterodimeric partner MAX, binds and recruits basal transcriptional machinery [33]. By comparing the promoter region of *LEF1* in different species, we found that human, rat, and mouse *LEF1* promoters contained 3 E-Box motifs in very conserved positions (Fig. 2i). We then used a bioinformatics approach to determine whether MYC has the ability to bind to the E-Boxes present within the *LEF1* promoter. We probed the Encyclopedia of DNA Elements (ENCODE) for chromatin immunoprecipitation (ChIP)-seq experiments using anti-MYC or MAX antibody and found that both MYC and MAX bound to E-Box 2 and E-Box 3 of *LEF1* in two independent cells lines (Additional file 4: Figure S4D).

To determine whether MYC directly binds to the promoter of *LEF1* in colon cancer cells, we performed ChIP for MYC in DLD1 colon cancer cells and determined that MYC bound strongly to the conserved E-Box 1 motif present in the *LEF1* promoter, but not to E-boxes 2 and 3 or to a non-specific control region 2.6 Kb upstream of *LEF1* gene, which we labelled as negative control (NC) (Fig. 2j). These results suggest that *LEF1* is a direct transcriptional target of MYC in colon cancer cells. However, we cannot exclude the possibility that additional transcription factors regulated downstream of MYC also contribute to LEF1 induction in MYC-transformed cells.

### Induction of LEF1 by MYC increases the nuclear pool of β-catenin

**Fig. 3:**
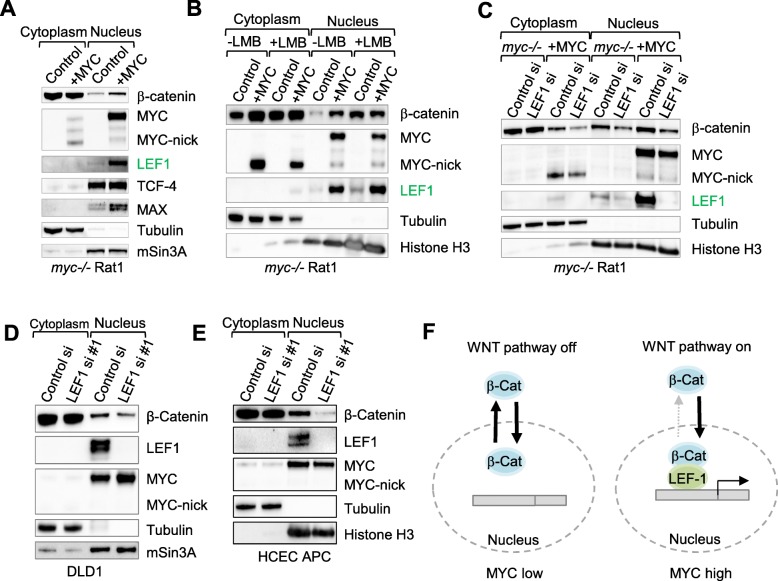
LEF1 increases nuclear retention of β-catenin in MYC-overexpressing cells. **a** Rat1 *myc−/−* fibroblasts with or without MYC expression were fractionated into cytosolic and nuclear fractions, and the samples were subjected to Western blotting with the indicated antibodies. **b** Rat1 fibroblasts were incubated with 20 μg/mL Leptomycin B for 3 h before fractionation and Western blotting with the indicated antibodies. **c**-**e** Rat1 fibroblasts (**c**), DLD1 cells (**d**) and HCEC cells with APC truncation mutation (**e**) were transfected with either control or LEF1 siRNA, and then fractionated for Western blotting with the indicated antibodies. **f** Model of activation of β-catenin by LEF1 in MYC-expressing cells

Many sporadic colon cancers contain activating mutations in the GTPase K-RAS, EGF (epidermal growth factor), and VEGF (vascular endothelial growth factor), which enhance canonical WNT signaling by increasing the concentration of β-catenin in the nucleus [34]. To determine whether MYC expression leads to an increase in the nuclear pool of β-catenin, we fractionated cells with or without MYC overexpression into cytosolic and nuclear fractions and found that MYC overexpression in both Rat1 fibroblasts and human epithelial cells increased nuclear β-catenin (Fig. 3a and Additional file 5: Figure S5A).

Preventing CRM1-dependent nuclear export through treatment with leptomycin B (LMB) caused the accumulation of β-catenin in the nucleus of control cells to the same extent as in MYC-expressing cells (Fig. 3b). However, LMB had no additional effect on the nuclear pool of β-catenin in MYC-expressing cells (Fig. 3b), thus suggesting that MYC-expressing cells display decreased nuclear export of β-catenin. LEF1 expression was previously shown to increase the nuclear pool of β-catenin, leading to its binding to DNA and transcriptional activation of WNT target genes [35]. Importantly, LEF1 knockdown prevented the nuclear accumulation of β-catenin in both control and MYC-expressing cells, indicating that LEF1 controls nuclear retention of β-catenin downstream of MYC (Fig. 3c). Knocking down LEF1 had no effect on subcellular localization of the β-catenin-related protein β-catenin (plakoglobin) (Additional file 5: Figure S5A), thus indicating that the nuclear retention of β-catenin by LEF1 is specific. Moreover, nuclear internalization of β-catenin upon MYC expression is specifically reduced by LEF1 knockdown; other MYC-induced transcription factors such as AHR did not affect β-catenin localization (Additional file 5: Figure S5B). These data are in agreement with data showing that LEF1 increases nuclear retention of β-catenin and increases its DNA binding and target gene activation [35, 36].

To determine whether LEF1 expression is necessary to maintain the nuclear pool of β-catenin in colon cancer cells, we silenced LEF1 in DLD1 cells and in HCEC partially transformed by truncated APC [25] (Additional file 4: Figure S4A). Nuclear and cytoplasmic fractionation experiments followed by Western blotting, confirmed that silencing LEF1 reduced the nuclear pool of β-catenin in the established colon cancer cell line DLD1 (Fig. 3d) and in HCEC-APC (Fig. 3e). Our results demonstrate that increased LEF1 expression by MYC led to the retention of β-catenin in the nucleus of normal and colon cancer cells (Fig. 3f).

### MYC-expressing cells are sensitive to inhibition of the WNT pathway

Because augmented LEF1 expression and nuclear retention of β-catenin in MYC- expressing cells suggest a potential increase in the activity of the WNT pathway, we first asked whether MYC expression can affect the activity of the WNT pathway measured by the activity of a reporter gene that contains TCF/LEF binding sites fused with luciferase (TOPFlash). To account for specificity of the WNT pathway, the activity values obtained for TOPFlash activity were normalized by the values obtained by a mutant form of this motif that cannot bind TCF/LEF factors named FOPFlash. This approach is the most well-established approach to globally measure transcriptional activity of the canonical WNT pathway [37].

**Fig. 4:**
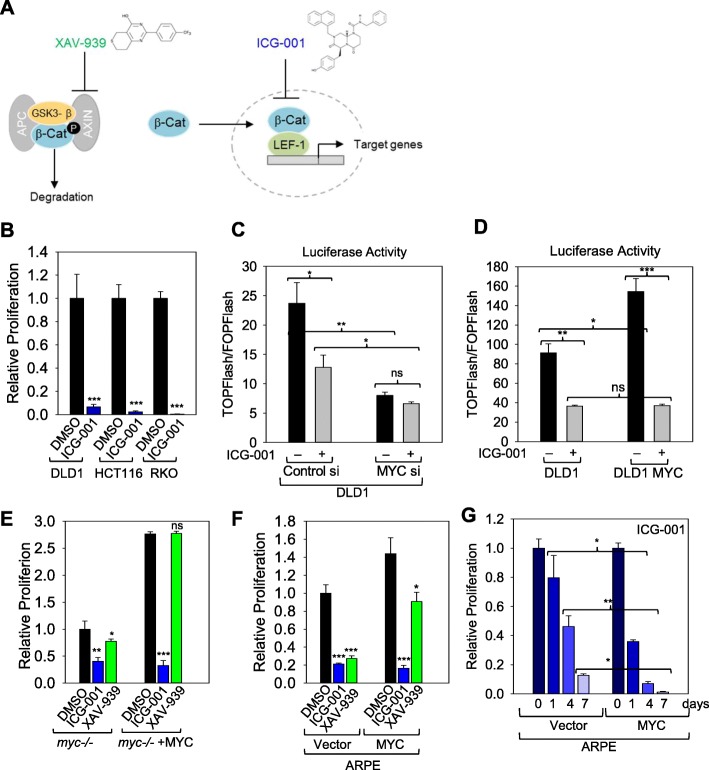
Cells expressing elevated levels of MYC are sensitive to inhibition of the WNT pathway. **a** Diagram of WNT pathway inhibitors, their structures, and targets. **b** 5000 DLD1, RKO, and HCT116 were plated separately in each well of 12-well plates and incubated with DMSO or ICG-001 in triplicate for 6 days. Cells were then stained with crystal violet, and the relative viabilities were qualified with ImageJ. **c** DLD1 cells were co-transfected with either control or MYC siRNA with a TOPFlash or FOPFlash luciferase reporter and treated with either DMSO or 20 μM ICG-001 overnight, and then luciferase activity was measured. **d** DLD1 cells with or without MYC overexpression were transfected with TOPFlash or FOPFlash luciferase reporter and treated with either DMSO or 20 μM ICG-001 overnight, then luciferase activity was measured. **e** 20,000 Rat1 *myc −/−* control fibroblast cells or Rat1 *myc −/−* cells reconstituted with human MYC were plated in 12-well plates and incubated with the indicated inhibitors in triplicate for 6 days. Cells were then stained with crystal violet, and the relative viabilities were qualified with ImageJ. **f** 5000 ARPE-19 cells with or without MYC overexpression were plated in 12-well plates and incubated with the indicated inhibitors in triplicate for 7 days. Cells were then stained with crystal violet, and the relative viabilities were qualified with ImageJ. **g** 5000 ARPE-19 cells with or without MYC overexpression were plated in 12-well plates and incubated with 20 μM ICG-001 in triplicate for 1–7 days. Cells were then stained with crystal violet, and the relative viabilities were qualified with ImageJ and all samples are normalized to 0-day sample of each cell line. * *p* < 0.05, ** *p* < 0.01, *** *p* < 0.001, ns not significant

To limit the WNT pathway activity without affecting the expression or localization of β-catenin and TCF/LEF factors, we used the inhibitor ICG-001. ICG-001 inhibits TCF/β-catenin–mediated transcription by binding to CBP, preventing its interaction with β-catenin and thus inhibits the canonical WNT pathway (Fig. 4a) [38, 39]. Indeed, treating colon cancer cell lines such as DLD1, HCT116, and RKO with the inhibitor ICG-001, which prevents the transcriptional activity of β-catenin, caused cell death (Fig. 4b), as previously reported [39]. Using TOPFlash/FOPFlash ratio to examine the effect of MYC on the activity of the WNT pathway, we found that DLD1 colon cancer cells transfected with TOPFlash reporter vector displayed constitutive TOPFlash activity that was reduced by treating cells with the inhibitor of the WNT pathway ICG-001 (Fig. 4c). Knocking down MYC caused a reduction in TOPFlash activity that was comparable to the reduction caused by ICG-001 (Fig. 4c). However, ICG-001 did not significantly alter TOPFlash activity in the cells transfected with MYC siRNA (Fig. 4c), which display reduced LEF1 expression. Overexpression of MYC in DLD1 cells increased activation of TOPFLash, and this activation was abrogated by treating cells with ICG-001 (Fig. 4d), thus demonstrating that TOPFlash activation by MYC is dependent on the WNT pathway.

To determine whether MYC-transformed cells are sensitive to the inhibition of the WNT pathway we treated fibroblasts and epithelial cells expressing either empty vector or MYC with ICG-001 and with another inhibitor of the WNT pathway named XAV-939. XAV-939 is a potent tankyrase inhibitor and stabilizes AXIN (Fig. 4a), resulting in the increased degradation of β-catenin [40]. The growth of Rat1 *myc−/−* cells expressing empty vector was only modestly inhibited by ICG-001, but not significantly affected by XAV-939 (Fig. 4e). In contrast, ICG-001 dramatically reduced the viability of Rat1 *myc−/−* cell reconstituted with MYC (Fig. 4e). Using the same inhibitors, we treated ARPE-19 cells expressing either empty vector or MYC and found that proliferation of MYC-expressing cells was more dramatically affected by ICG-001 than by XAV-939 (Fig. 4f). A time course experiment with ICG-001 in ARPE-19 cells expressing either empty vector or MYC demonstrated that ICG-001 caused cell death more quickly in MYC-expressing cells than in control cells (Fig. 4g).

MYC-expressing cells not only died faster but also were more sensitive to lower amounts of ICG-001 (Additional file 6: Figure S6B). Based on these results, we concluded that MYC-driven cell proliferation requires an active WNT pathway and that repressing WNT pathway activity particularly by directly interfering with the transcriptional activity of the WNT pathway caused apoptosis preferentially of MYC-expressing cells. Our results indicate that cells expressing elevated levels of MYC are dependent on the LEF1/β-catenin complex to sustain rapid proliferation.

### LEF1 knockdown leads to apoptosis of MYC-expressing cells and colon cancer cells

**Fig. 5:**
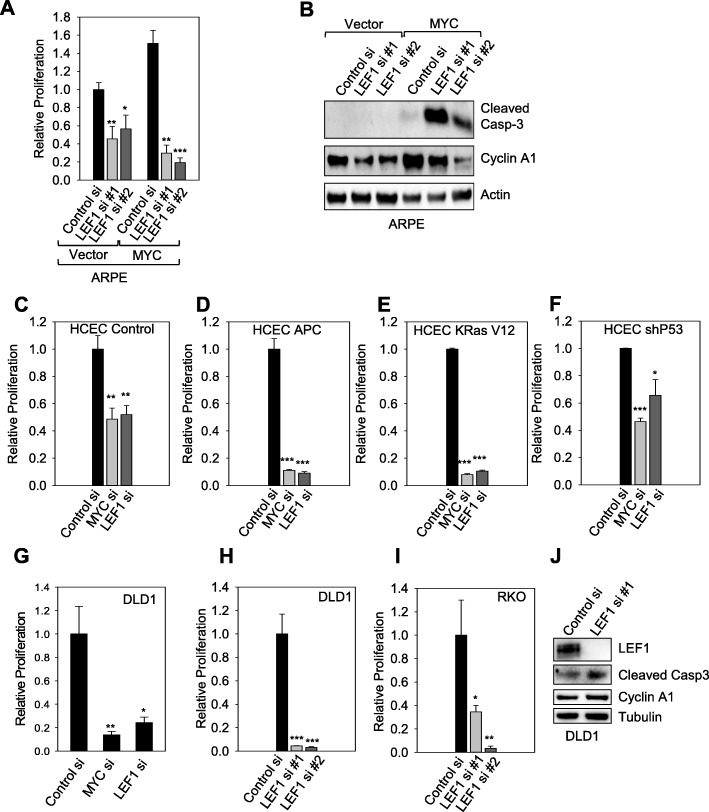
Cells expressing elevated levels of MYC are sensitive to LEF1 knockdown. **a** 5000 ARPE-19 cells with or without MYC overexpression were transfected with either control or LEF1 siRNA in 12-well plates for 7 days. Then the cells were stained with crystal violet, and the cell densities were quantified by using ImageJ. **b** ARPE-19 cells were extracted with NP40 lysing buffer, and the total cell lysates were subjected to Western blotting with the indicated antibodies. **c**-**f** 20,000 HCEC cells expressing control vector (**c**), truncated APC (**d**), KRas V12 (E), or P53 shRNA (F) were transfected with control, MYC, or LEF1 siRNA in 12-well plates for 7 days. Then the cells were stained with crystal violet, and the cell densities were quantified by using ImageJ. **g** 5000 DLD1 cells were transfected with control, MYC, or LEF1 siRNA in 12-well plates for 7 days, when cells were stained with crystal violet, and the cell densities were quantified by using ImageJ. **h**, **i** 5000 DLD1 (**h**) or RKO cells (**i**) were transfected with control or LEF1 siRNA in 12-well plates for 7 days. Then the cells were stained with crystal violet, and the cell densities were quantified by using ImageJ. **j** DLD1 cells were transfected with either control or LEF1 siRNAs for 3 days and then extracted with NP40 lysing buffer and subjected to Western blotting. * *p* < 0.05, ** *p* < 0.01, *** *p* < 0.001

MYC-expressing cells were sensitive to inhibitors of the transcriptional activity of the WNT pathway. To determine whether LEF1 activity was required for the proliferation of MYC-expressing cells, we performed siRNA-mediated knockdown for LEF1 by transfecting two independent siRNAs in ARPE-19 cells expressing either empty vector or MYC (Additional file 6: Figure S6C). We quantified the relative cell viability 7 days after LEF1 knockdown and found a dramatic reduction in the viability of cells overexpressing MYC (Fig. 5a). More modest effects on cell viability were observed with cells expressing empty vector upon LEF1 knockdown (Fig. 5a). LEF1 knockdown decreased the expression of Cyclin A, a cell cycle marker in both control and MYC-expressing cells (Fig. 5b), thus suggesting that loss of LEF1 affects cell proliferation independent of MYC expression. However, LEF1 knockdown caused a dramatic increase in the levels of cleaved caspase 3 in MYC-expressing cells but not in cell expressing the empty vector, thus demonstrating that loss of LEF1 leads to apoptosis specifically of MYC-expressing cells (Fig. 5b).

Colon cancer is initiated by the inactivation of APC, which leads to the activation of the WNT pathway and the development of colon cancer in a MYC-dependent manner [15]. Moreover tumors initiated by the activating mutation in KRAS also display a dependency on MYC to grow [41]. Given that APC loss of function and KRAS hyperactivation are part of the initial steps in colon cancer formation (Additional file 4: Figure S4A), we asked whether LEF1 is required for the growth of HCEC partially transformed by the expression of truncated APC, mutant KRAS (V12) or knocked down for P53, the 3 major genetic events driving colon cancer formation. We found that both MYC and LEF1 silencing reduced viability of HCEC expressing mutant KRAS or truncated APC more dramatically than control HCEC or HCEC infected with shRNA for P53 (Fig. 5 c-f). We also compared the effects of MYC and LEF1 silencing in DLD1 cells and found similar reduction in cell proliferation (Fig. 5g). Hence, our results suggest that MYC-dependent colon cancer cell are specifically sensitive to reduction in LEF1 expression.

Knocking down MYC or LEF1 caused a dramatic reduction in the viability of the colon cancer cells DLD1 and RKO (Fig. 5 h, i). Indeed, LEF1 knockdown was previously shown to cause a reduction in colon cancer cell growth in vitro and when xenotransplanted into immune-deficient mice [12]. Similar to the results obtained for fibroblasts (Fig. 5b), LEF1 knockdown led to increases in the levels of cleaved caspase 3, an indication of apoptosis and cell cycle arrest, but not in Cyclin D1(Fig. 5j). Silencing LEF1 had variable effects on the expression of endogenous MYC depending on the cell line used for the experiment. For example, silencing LEF1 in RKO cells did not affect MYC levels dramatically; silencing LEF1 significantly reduced MYC levels in Huh7 cells (Additional file 6: Figure S6A), indicating that other pathways contribute to MYC expression in fully transformed cells (Additional file 6: Figure S6A).

### LEF1 regulates metabolic pathways downstream of MYC

MYC is a master regulator of cellular metabolism, and the genes transcriptionally regulated by MYC are critical for the metabolic reprograming of cancer cells [42]. Given that LEF1 is necessary for the proliferative phenotype of cells overexpressing MYC, we interrogated the contribution of LEF1 to the metabolism of MYC-expressing cells. We performed metabolomics analyses comparing *myc−/−* cells reconstituted with MYC and transfected with either siControl or siLEF1. Metabolomics were performed two days after siRNA transfection when silencing was effective and MYC-expressing cells were still viable (Additional file 7: Figure S7A). As expected, LEF1 silencing had no effect on the expression of exogenous MYC in these cells (Additional file 7: Figure S7B). Results of triplicate samples (Additional file 7: Figure S7C) were obtained using a cut off of 30% changes and VIP score above 1; LEF1 knockdown altered the levels of 32 metabolites in MYC-expressing cells (Additional file 8: Figure S8A). Among the metabolic changes observed upon LEF1 knockdown in MYC-expressing cells were amino acids, component of the TCA cycle, purines, and lipids (Additional file 8: Fig. S8B). Interestingly, several amino acids including arginine, tryptophan, tyrosine and histidine were elevated upon LEF1 knockdown probably due to the reduced utilization of amino acids in slowly proliferating cells (Additional file 8: Fig. S8B).

**Fig. 6:**
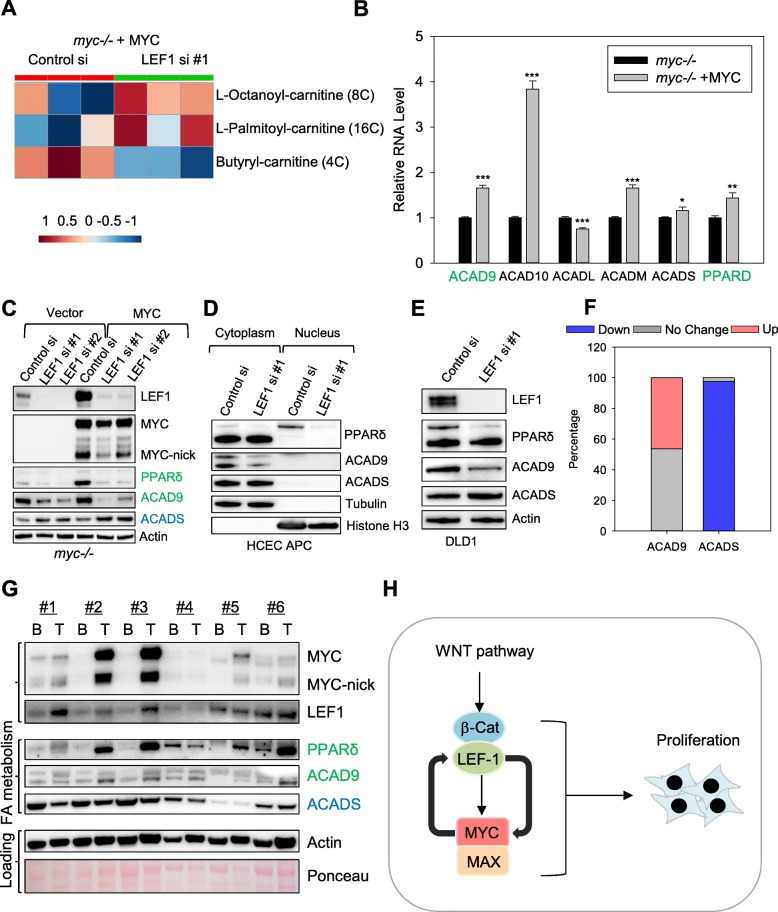
LEF1 regulates lipid metabolism in MYC-expressing cells. **a** Heatmap of acylcarnitines affected by LEF1 siRNA in *myc−/−* + MYC fibroblast, generated by metabolomics analyses of cells transfected with either control or LEF1 siRNA. **b** Rat1 *myc−/−* fibroblasts expressing either vector or human MYC were collected and subjected for RNA-seq analysis and expression levels of genes involving lipid metabolism were compared. **c** Rat1 *myc −/−* control fibroblast cells and Rat1 *myc −/−* cells reconstituted with human MYC were transfected with control or LEF1 siRNAs for 3 days and extracted with NP40 lysing buffer for Western blot assays with the indicated antibodies. **d** HCEC expressing truncated APC were transfected with either control or LEF1 siRNA for 3 days, and then fractionated into nuclear and cytoplasmic fractions for Western blotting with the indicated antibodies. **e** DLD1 cells transfected with control of LEF1 siRNAs for three days were extracted with NP40 lysis buffer, and the total lysates were subjected to Western blot assays. **f** ACAD9 and CADS gene expression in colon cancer and adjacent normal tissues deposited in the TCGA database. Blue: downregulated (< 0.7), grey: no change (0.7–1.3), and red: upregulated (> 1.3). *n* = 41 pairs. **g** Human colon tumor tissues (T) and their surrounding benign tissues (B) were extracted with NP40 lysis buffer, and the total lysates were subjected to Western blotting with the indicated antibodies. **h** Model of MYC and LEF1 positive feedback loop in proliferating colonic cells

We then further investigated the regulation of fatty acid metabolism by LEF1 in MYC-expressing cells for two reasons: first, previous studies found that expression of a dominant negative LEF1 variant in the skin leads to changes in lipid composition marked by an increase in long chain fatty acids-containing ceramide in the skin of these mice [43]; second, our RNA-seq revealed that MYC regulated the expression of genes involved in fatty acid oxidation (Fig. 6b).

Our metabolomics analyses (Additional file 8: Figure S8) on *myc−/−* cells reconstituted with human MYC and transfected with either control or Lef1 siRNA found that LEF1 silencing in MYC-expressing cells led to the accumulation of long-chain fatty acid (LCFA) L-palmitoylcarnitine and medium chain fatty acid (MCFA) L-Octanoylcarnitine (Fig. 6a, Additional file 8: Figure S8C). Butyrylcarnitine, which is a SCFA with 4 carbons that can be generated from longer fatty acids was lower in MYC-expressing cells when LEF1 was knocked down (Fig. 6a, Additional file 8: Figure S8C).

### LEF1 is necessary for the expression of PPARδ and ACAD9 downstream of MYC

Our RNA-seq data revealed that MYC expression led to an increase in the expression of peroxisome proliferator-activated receptor δ (*PPARD)* (Fig. 6b). PPARδ regulates the expression of genes that transport long-chain fatty acids into the mitochondria for β-oxidation [44] as a master regulator of fatty acid metabolism. The reduction in PPARδ upon LEF1 knockdown could cause a defect in the import of fatty acids into the mitochondria, thus delaying their processing, leading to the accumulation of MCFA and LCFA such as L-palmitoylcarnitine and L-Octanoylcarnitine (Fig. 6a).

PPARδ is upregulated in human colorectal polyps and colorectal cancers [45] and has been identified as a downstream target of β-catenin [46]. While the link between the WNT pathway and PPARδ has been controversial [47], recently, a high-fat diet was reported to increase PPARδ activity in progenitor intestinal cells of animals with APC-inactivating mutations [44]. We confirmed the induction of PPARδ by MYC overexpression in multiple cell lines (Fig. 1c-e). Conversely knocking down MYC led to a downregulation in PPARδ (Fig. 2 d, e). To determine whether the upregulation of PPARδ in MYC-expressing cells was dependent on the activation of the WNT pathway caused by LEF1 expression, we knocked down LEF1 in *myc−/−* fibroblast expressing either empty vector or reconstituted with MYC. We found that PPARδ expression was reduced by LEF1 knockdown in both cell lines (Fig. 6c).

Because LEF1 is required for the expression of PPARδ in MYC-expressing cells, we examined the regulation of other fatty acid-regulating genes by MYC (Fig. 6b). *We found that the mRNA for the Acyl CoA dehydrogenases ACAD9, ACAD10, and ACADM were also induced by MYC (*Fig. *6**b).* Fatty acid metabolism requires the activity of ACADs including ACADS, ACADM, ACADL, ACADVL, ACAD9, and ACAD10, which are specialized for short-, medium-, long- and very long chain fatty acids [48]. LEF1 knockdown specifically reduced ACAD9 levels, similarly to PPARδ, both in MYC-expressing fibroblast as well as in *myc−/−* cells (Fig. 6c). ACAD10 expression was not validated, and ACADS was elevated upon LEF1 siRNA (Fig. 6c). The increase in ACADS was likely to be responsible, at least in part, for the increase in butyrylcarnitine upon LEF1 knockdown.

LEF1 knockdown also caused reductions in the expression of PPARδ and ACAD9 in colonic cells (Fig. 6d, e). Western blotting of fractionated HCEC-APC samples showed that ACAD9 level was also reduced by LEF1 knockdown, and most interestingly, only nuclear, therefore active PPARδ was decreased by LEF1 siRNA (Fig. 6d). LEF1 was also necessary for maximum expression of PPARδ and ACAD9 in the fully transformed DLD1 colon cancer cells (Fig. 6e). The expression of MYC, LEF1, PPARδ, and ACAD9 were closely correlated in a Western blot comparing colon cancer and normal adjacent benign tissue of human samples (Fig. 6g). ACADS expression was inversely correlated with MYC and LEF1 (Fig. 6g), similarly to the expression pattern obtained from fibroblasts (Fig. 6c). Data from TCGA database analyses also revealed that approximately 50% of colon cancer patients had elevated ACAD9 mRNA levels, while the majority of patients displayed a reduction in ACADS mRNA (Fig. 6f). Previous metabolomics study discovered that palmitate and oleate levels increase in MYC-driven colorectal tumors, while enzymes involving fatty acid oxidation, including ACADS, are down-regulated [49].

In summary, we found that LEF1 is induced downstream of MYC and activates the expression of genes such as ACAD9 and PPARδ in MYC-expressing cells. Additional genes regulated by LEF1 in MYC-transformed cells may be involved in other metabolic and non-metabolic pathways that are important for the deregulated proliferation or other aspects of cancer cell fitness.

## Discussion

Our results indicate that MYC induces the expression of LEF1 and that LEF1, by interacting with β-catenin within nucleus, activates the expression of PPARδ and ACAD9. Because PPARδ and ACAD9 are required for fatty acid metabolism, reduction in their levels upon LEF1 knockdown may cause accumulation of unprocessed fatty acids which may contribute to apoptosis of MYC-expressing cells. Interestingly, β-catenin was recently shown to be necessary for the expression of ACADL, ACADVL, and ACADS in osteoclasts [50]. Regulation of ACAD9 and PPARδ by LEF1 underscores the importance of future studies to identify the global signature of genes regulated by LEF1 and potentially other TCF factors in normal and MYC-dependent colonic cells to define the molecular crosstalk between MYC and the WNT pathway in colon cancer. Indeed, our metabolomics data suggest that MYC-dependent LEF1 expression might also regulate cell proliferation by altering other metabolic pathways like the TCA cycle and amino acids metabolism (Additional file 8: Figure S8B), which warrant further studies.

While WNT pathway activation is unquestionably a major driver in the development of colonic adenomas, studies have shown that activation of additional signaling pathways are required for optimal nuclear localization of β-catenin in colon cancer cells. For example, *KRAS* induces the phosphorylation of the Frizzled co-receptor LRP6, leading to a cascade of events that promotes activation of the WNT pathway and increases in cell migration and tumor growth [51]. MYC was also shown to regulate genes that lead to the activation of the WNT pathway. MYC was found to repress the WNT inhibitors DKK1 and SFRP1, leading to the activation of the WNT pathway in breast cancer cell lines [52]. Moreover, a recent study proposed that MYC expression leads to global increase in mRNA cap methylation including mRNAs that encode proteins in the WNT pathway, such as *GSK3β, APC, LRP5, CTNNB1*, and *TCF7*, and results in their increased translation [53]. These studies, combined with our current results showing MYC modulates the transcription of genes in the WNT pathway, indicate that regulation of the WNT pathway by MYC involves multiple mechanisms and may be a widespread event in transformed cells. Therefore, we propose a model for MYC and WNT cooperation in tumors (Fig. 6h). In our putative model MYC and LEF1 engage in a positive feedback loop in which oncogenic hits that activate the WNT pathway induce MYC expression in cancer cells. MYC, in turn, directly promotes transcription of LEF1 which further potentiates the WNT pathway activation.

## Conclusions

Our study demonstrates that MYC regulated the expression of the β-catenin co-transcriptional factor LEF1. MYC expression caused the retention of β-catenin in the nucleus in a LEF1-dependent manner and resulted in the activation of WNT target genes. Moreover, MYC-expressing cells became dependent on LEF1 activity to proliferate. Therefore, we identified a novel pathway by which MYC induces the expression of LEF1 to activate the WNT pathway, which is required for the hyperproliferative behavior of MYC-transformed cells.

## Supplementary information

**Additional file 1: Figure S1.** (A) Major groups of genes in the WNT pathway are regulated by MYC. Red color represents activated genes, blue repressed genes and purple represents families of genes that contain members repressed and activated by MYC. (B) RT-qPCR for the indicated genes in Rat1 fibroblasts *myc*−/− or Rat1 fibroblasts *myc*−/− reconstituted with human MYC. Expression levels of each gene was normalized to the levels of 18S, and the expression levels of each genes in *myc*−/− samples was set to 1. (C) RT-qPCR for the indicated genes in ARPE-19 cells stably expressing empty vector or MYC. Expression levels of each gene was normalized to the levels of 18S, and the expression levels of each genes in ARPE control samples was set to 1. (D) Rat1 fibroblasts wild type (WT), *myc−/−* stably expressing empty vector or MYC were extracted with NP40 lysing buffer, and the total cell lysates were subjected to Western blotting with the indicated antibodies. (E) ARPE-19 cells stably expressing empty vector or MYC were extracted with NP40 lysing buffer, and the total cell lysates were subjected to Western blotting with the indicated antibodies. (F) DLD1 cells were transfected with control, MYC, or AHR siRNAs for 3 days before total protein extraction and Western blot assays. * *p* < 0.05, ** *p* < 0.01, *** *p* < 0.001.

**Additional file 2: Figure S2.** (A) Comparison of the levels of the indicated genes in cancer tissues and adjacent normal tissues from TCGA database. Blue: downregulated (< 0.7), grey: no change (0.7–1.3), and red: upregulated (> 1.3). *n* = 41 pairs. (B) Kaplan–Meier curves comparing survival of patients with colon adenocarcinoma (COAD) divided into high and low expression levels of MYC-driven WNT signaling genes. Comparison between 30% highest and 30% lowest expression were generated using ONCLnc (http://www.oncolnc.org/).

**Additional file 3: Figure S3.** (A) Gene levels of MYC-driven WNT signaling genes were compared between cancer tissues and normal tissues, and binary heatmaps were drawn to show the changes. Samples were sorted with *p*-value, and changes with p-value< 0.05 were considered statistically significant. (B) Kaplan–Meier curves comparing survival of various cancer types correlated with LEF1. Comparisons between 30% highest and 30% lowest expression were generated using ONCLnc (http://www.oncolnc.org/).

**Additional file 4: Figure S4.** (A) Scheme of colon cancer progression. (B) Genetic background of colonic cells used in this study. Information of cancer cell was obtained from Colorectal Cancer Atlas (http://colonatlas.org/). (C) DLD1 cells were transfected with either control or MYC siRNAs, and the relative RNA levels were measured by using RT-qPCR. (D) ChIP-seq data from the ENCODE database show that MYC and MAX binds to the LEF1 promoter in K562 and MCF10A cells. Cluster Scores (out of 1000) are shown in parentheses. (E) Rat fibroblast expressing inducible MYC expression (Tet-On) was cultured in the presence of 1 μg/ml doxycycline (Dox) for 48 h. Incubation with 25 μg/ml cycloheximide (CHX) for three hours was performed prior to RNA isolation and RT-qPCR analysis. Protein synthesis inhibition reduced LEF1 mRNA both in control and MYC expression cells, but to a less extent in MYC-expressing cells.

**Additional file 5: Figure S5.** (A) ARPE-19 cells with or without MYC overexpression were fractionated into cytosolic and nuclear fractions, and the samples were subjected to Western blotting with the indicated antibodies. (B) ARPE-19 cells with or without MYC overexpression were transfected with either control or AHR siRNA, and then fractionated for Western blotting with the indicated antibodies.

**Additional file 6: Figure S6.** (A) RKO and Huh7 cells were transfected with control or LEF1 siRNAs for 3 days and then Western blotted. (B) 5000 ARPE-19 cells with or without MYC overexpression were plated in 12-well plates and incubated with different concentrations of ICG-001 in triplicate for 7 days. Cells were then stained with crystal violet, and the relative viabilities were qualified with ImageJ. (C) RT-qPCR for LEF1 in APRE cells infected with empty vector transfected with LEF1 siRNA.

**Additional file 7: Figure S7.** (A) Diagram of the metabolomics experiment showing Rat1 *myc−/−* fibroblasts with human MYC expression were transfected with either control or LEF1 siRNAs in triplicate. Cells were extracted with methanol 2 days after transfection and processed for LC/MS metabolomics. (B) Parallel dishes from the same experiment were exacted with NP40 lysis buffer and subjected to Western blotting. (C) Principal component analysis (PCA) plot was generated by SIMCA 13.0.3 to show the sample clusters, and t [1] and t [2] are variances of the samples.

**Additional file 8: Figure S8.** (A) Heatmap comparing the metabolic profile of *myc−/−* cell reconstituted with MYC and transfected with either control siRNA or siRNA for LEF1. (B) Metabolites and metabolic pathways altered by LEF1 silencing. (C) Diagram of β-oxidation with metabolites affected by LEF1 knockdown in MYC-expressing cells and their corresponding enzymes.

**Additional file 9: Table S1.** List of primers.

## Data Availability

The datasets used and/or analyzed during the current study are available from the corresponding author on reasonable request.

## Abbreviations

ACAD9: Acyl CoA dehydrogenase 9
AHR: Aryl hydrocarbon receptor
APC: Adenomatous polyposis coli
DKK1: Dickkopf-related protein 1
DVL1/2: dishevelled ½
FZD2: Frizzled 2
HCEC: Human colonic epithelial cell
LEF1: Lymphoid enhancer binding factor 1
LIF: Leukemia inhibitory factor
LRP5/6: Low-density lipoprotein receptor-related protein 5/6
MAX: MYC-associated factor X
PPARδ: Peroxisome proliferator activated receptor delta
TCF: Transcription factor

## References

[1] Lao VV, Grady WM (2011). Epigenetics and colorectal cancer. Nat Rev Gastroenterol Hepatol.

[2] Fearon ER, Vogelstein B (1990). A genetic model for colorectal tumorigenesis. Cell.

[3] Jasperson KW, Tuohy TM, Neklason DW, Burt RW (2010). Hereditary and familial colon cancer. Gastroenterology.

[4] MacDonald BT, Tamai K, He X (2009). Wnt/beta-catenin signaling: components, mechanisms, and diseases. Dev Cell.

[5] Gross JC, Chaudhary V, Bartscherer K, Boutros M (2012). Active Wnt proteins are secreted on exosomes. Nat Cell Biol.

[6] Alexandre C, Baena-Lopez A, Vincent JP (2014). Patterning and growth control by membrane-tethered wingless. Nature.

[7] Basu Sayon, Haase Gal, Ben-Ze'ev Avri (2016). Wnt signaling in cancer stem cells and colon cancer metastasis. F1000Research.

[8] Najdi R, Holcombe RF, Waterman ML (2011). Wnt signaling and colon carcinogenesis: beyond APC. J Carcinog.

[9] Clevers H, Loh KM, Nusse R (2014). Stem cell signaling. An integral program for tissue renewal and regeneration: Wnt signaling and stem cell control. Science.

[10] Stamos JL, Weis WI (2013). The beta-catenin destruction complex. Cold Spring Harb Perspect Biol.

[11] Hovanes K, Li TW, Munguia JE, Truong T, Milovanovic T, Lawrence Marsh J, Holcombe RF, Waterman ML (2001). Beta-catenin-sensitive isoforms of lymphoid enhancer factor-1 are selectively expressed in colon cancer. Nat Genet.

[12] Wang WJ, Yao Y, Jiang LL, Hu TH, Ma JQ, Liao ZJ, Yao JT, Li DF, Wang SH, Nan KJ (2013). Knockdown of lymphoid enhancer factor 1 inhibits colon cancer progression in vitro and in vivo. PLoS One.

[13] Shtutman M, Zhurinsky J, Simcha I, Albanese C, D'Amico M, Pestell R, Ben-Ze'ev A (1999). The cyclin D1 gene is a target of the beta-catenin/LEF-1 pathway. Proc Natl Acad Sci U S A.

[14] He TC, Sparks AB, Rago C, Hermeking H, Zawel L, da Costa LT, Morin PJ, Vogelstein B, Kinzler KW (1998). Identification of c-MYC as a target of the APC pathway. Science.

[15] Sansom OJ, Meniel VS, Muncan V, Phesse TJ, Wilkins JA, Reed KR, Vass JK, Athineos D, Clevers H, Clarke AR (2007). Myc deletion rescues Apc deficiency in the small intestine. Nature.

[16] Dang CV (2015). Web of the extended Myc network captures metabolism for tumorigenesis. Cancer Cell.

[17] Hsieh AL, Walton ZE, Altman BJ, Stine ZE, Dang CV (2015). MYC and metabolism on the path to cancer. Semin Cell Dev Biol.

[18] Kress TR, Sabo A, Amati B (2015). MYC: connecting selective transcriptional control to global RNA production. Nat Rev Cancer.

[19] Hatton KS, Mahon K, Chin L, Chiu FC, Lee HW, Peng D, Morgenbesser SD, Horner J, DePinho RA (1996). Expression and activity of L-Myc in normal mouse development. Mol Cell Biol.

[20] Brodeur GM, Seeger RC, Schwab M, Varmus HE, Bishop JM (1984). Amplification of N-myc in untreated human neuroblastomas correlates with advanced disease stage. Science.

[21] Nau MM, Brooks BJ, Battey J, Sausville E, Gazdar AF, Kirsch IR, McBride OW, Bertness V, Hollis GF, Minna JD (1985). L-myc, a new myc-related gene amplified and expressed in human small cell lung cancer. Nature.

[22] Beroukhim R, Mermel CH, Porter D, Wei G, Raychaudhuri S, Donovan J, Barretina J, Boehm JS, Dobson J, Urashima M (2010). The landscape of somatic copy-number alteration across human cancers. Nature.

[23] Liu W, Le A, Hancock C, Lane AN, Dang CV, Fan TW, Phang JM (2012). Reprogramming of proline and glutamine metabolism contributes to the proliferative and metabolic responses regulated by oncogenic transcription factor c-MYC. Proc Natl Acad Sci U S A.

[24] Yochum GS, Sherrick CM, Macpartlin M, Goodman RH (2010). A beta-catenin/TCF-coordinated chromatin loop at MYC integrates 5′ and 3′ Wnt responsive enhancers. Proc Natl Acad Sci U S A.

[25] Roig AI, Eskiocak U, Hight SK, Kim SB, Delgado O, Souza RF, Spechler SJ, Wright WE, Shay JW (2010). Immortalized epithelial cells derived from human colon biopsies express stem cell markers and differentiate in vitro. Gastroenterology.

[26] Schneider CA, Rasband WS, Eliceiri KW (2012). NIH image to ImageJ: 25 years of image analysis. Nat Methods.

[27] Korinek V, Barker N, Morin PJ, van Wichen D, de Weger R, Kinzler KW, Vogelstein B, Clevers H (1997). Constitutive transcriptional activation by a beta-catenin-Tcf complex in APC−/− colon carcinoma. Science.

[28] Anso E, Mullen AR, Felsher DW, Mates JM, Deberardinis RJ, Chandel NS (2013). Metabolic changes in cancer cells upon suppression of MYC. Cancer Metab.

[29] Mullen AR, Hu Z, Shi X, Jiang L, Boroughs LK, Kovacs Z, Boriack R, Rakheja D, Sullivan LB, Linehan WM (2014). Oxidation of alpha-ketoglutarate is required for reductive carboxylation in cancer cells with mitochondrial defects. Cell Rep.

[30] Lafita-Navarro MC, Kim M, Borenstein-Auerbach N, Venkateswaran N, Hao YH, Ray R, Brabletz T, Scaglioni PP, Shay JW, Conacci-Sorrell M (2018). The aryl hydrocarbon receptor regulates nucleolar activity and protein synthesis in MYC-expressing cells. Genes Dev.

[31] Patel RK, Jain M (2012). NGS QC toolkit: a toolkit for quality control of next generation sequencing data. PLoS One.

[32] Love MI, Huber W, Anders S (2014). Moderated estimation of fold change and dispersion for RNA-seq data with DESeq2. Genome Biol.

[33] Blackwood EM, Eisenman RN (1991). Max: a helix-loop-helix zipper protein that forms a sequence-specific DNA-binding complex with Myc. Science.

[34] Wu X, Tu X, Joeng KS, Hilton MJ, Williams DA, Long F (2008). Rac1 activation controls nuclear localization of beta-catenin during canonical Wnt signaling. Cell.

[35] Jamieson C, Sharma M, Henderson BR (2011). Regulation of beta-catenin nuclear dynamics by GSK-3beta involves a LEF-1 positive feedback loop. Traffic.

[36] Morgan RG, Ridsdale J, Payne M, Heesom KJ, Wilson MC, Davidson A, Greenhough A, Davies S, Williams AC, Blair A, et al. LEF-1 drives aberrant beta-catenin nuclear localization in myeloid leukemia cells. Haematologica. 2019. 10.3324/haematol.2018.202846 PMC6601079 30630973

[37] Veeman MT, Slusarski DC, Kaykas A, Louie SH, Moon RT (2003). Zebrafish prickle, a modulator of noncanonical Wnt/Fz signaling, regulates gastrulation movements. Curr Biol.

[38] Grigson ER, Ozerova M, Pisklakova A, Liu H, Sullivan DM, Nefedova Y (2015). Canonical Wnt pathway inhibitor ICG-001 induces cytotoxicity of multiple myeloma cells in Wnt-independent manner. PLoS One.

[39] Emami KH, Nguyen C, Ma H, Kim DH, Jeong KW, Eguchi M, Moon RT, Teo JL, Kim HY, Moon SH (2004). A small molecule inhibitor of beta-catenin/CREB-binding protein transcription [corrected]. Proc Natl Acad Sci U S A.

[40] Huang SM, Mishina YM, Liu S, Cheung A, Stegmeier F, Michaud GA, Charlat O, Wiellette E, Zhang Y, Wiessner S (2009). Tankyrase inhibition stabilizes axin and antagonizes Wnt signalling. Nature.

[41] Soucek L, Whitfield JR, Sodir NM, Masso-Valles D, Serrano E, Karnezis AN, Swigart LB, Evan GI (2013). Inhibition of Myc family proteins eradicates KRas-driven lung cancer in mice. Genes Dev.

[42] Dang C. V. (2013). MYC, Metabolism, Cell Growth, and Tumorigenesis. Cold Spring Harbor Perspectives in Medicine.

[43] Fehrenschild D, Galli U, Breiden B, Bloch W, Schettina P, Brodesser S, Michels C, Gunschmann C, Sandhoff K, Niessen CM (2012). TCF/Lef1-mediated control of lipid metabolism regulates skin barrier function. J Invest Dermatol.

[44] Beyaz S, Mana MD, Roper J, Kedrin D, Saadatpour A, Hong SJ, Bauer-Rowe KE, Xifaras ME, Akkad A, Arias E (2016). High-fat diet enhances stemness and tumorigenicity of intestinal progenitors. Nature.

[45] Takayama O, Yamamoto H, Damdinsuren B, Sugita Y, Ngan CY, Xu X, Tsujino T, Takemasa I, Ikeda M, Sekimoto M (2006). Expression of PPARdelta in multistage carcinogenesis of the colorectum: implications of malignant cancer morphology. Br J Cancer.

[46] He TC, Chan TA, Vogelstein B, Kinzler KW (1999). PPARdelta is an APC-regulated target of nonsteroidal anti-inflammatory drugs. Cell.

[47] Lecarpentier Y, Claes V, Vallee A, Hebert JL (2017). Interactions between PPAR gamma and the canonical Wnt/Beta-catenin pathway in type 2 diabetes and Colon Cancer. PPAR Res.

[48] Houten SM, Wanders RJ (2010). A general introduction to the biochemistry of mitochondrial fatty acid beta-oxidation. J Inherit Metab Dis.

[49] Satoh K, Yachida S, Sugimoto M, Oshima M, Nakagawa T, Akamoto S, Tabata S, Saitoh K, Kato K, Sato S (2017). Global metabolic reprogramming of colorectal cancer occurs at adenoma stage and is induced by MYC. Proc Natl Acad Sci U S A.

[50] Frey JL, Kim SP, Li Z, Wolfgang MJ, Riddle RC (2018). Beta-catenin directs Long-chain fatty acid catabolism in the osteoblasts of male mice. Endocrinology.

[51] Cagnol S, Rivard N (2013). Oncogenic KRAS and BRAF activation of the MEK/ERK signaling pathway promotes expression of dual-specificity phosphatase 4 (DUSP4/MKP2) resulting in nuclear ERK1/2 inhibition. Oncogene.

[52] Cowling VH, D'Cruz CM, Chodosh LA, Cole MD (2007). C-Myc transforms human mammary epithelial cells through repression of the Wnt inhibitors DKK1 and SFRP1. Mol Cell Biol.

[53] Posternak V, Ung MH, Cheng C, Cole MD (2017). MYC mediates mRNA cap methylation of canonical Wnt/beta-catenin signaling transcripts by recruiting CDK7 and RNA methyltransferase. Mol Cancer Res.

