# Supplementary material for: Induction of LEF1 by MYC activates the WNT pathway and maintains cell proliferation
Source: Cell Commun Signal. 2019 Oct 17;17:129. doi: 10.1186/s12964-019-0444-1 (PMC6798382; doi:10.1186/s12964-019-0444-1)
Supplement: Supplementary file 1 — Additional file 1: Figure S1. (A) Major groups of genes in the WNT pathway are regulated by MYC. Red color represents activated genes, blue repressed genes and purple represents families of genes that contain members repressed and activated by MYC. (B) RT-qPCR for the indicated genes in Rat1 fibroblasts myc−/− or Rat1 fibroblasts myc−/− reconstituted with human MYC. Expression levels of each gene was normalized to the levels of 18S, and the expression levels of each genes in myc−/− samples was set to 1. (C) RT-qPCR for the indicated genes in ARPE-19 cells stably expressing empty vector or MYC. Expression levels of each gene was normalized to the levels of 18S, and the expression levels of each genes in ARPE control samples was set to 1. (D) Rat1 fibroblasts wild type (WT), myc−/− stably expressing empty vector or MYC were extracted with NP40 lysing buffer, and the total cell lysates were subjected to Western blotting with the indicated antibodies. (E) ARPE-19 cells stably expressing empty vector or MYC were extracted with NP40 lysing buffer, and the total cell lysates were subjected to Western blotting with the indicated antibodies. (F) DLD1 cells were transfected with control, MYC, or AHR siRNAs for 3 days before total protein extraction and Western blot assays. * p < 0.05, ** p < 0.01, *** p < 0.001. [file 12964_2019_444_MOESM1_ESM.docx]

Additional file 1: **Figure S1.** (A) Major groups of genes in the WNT pathway are regulated by MYC. Red color represents activated genes, blue repressed genes and purple represents families of genes that contain members repressed and activated by MYC. (B) RT-qPCR for the indicated genes in Rat1 fibroblasts *myc*-/- or Rat1 fibroblasts *myc*-/- reconstituted with human MYC. Expression levels of each gene was normalized to the levels of 18S, and the expression levels of each genes in *myc*-/- samples was set to 1. (C) RT-qPCR for the indicated genes in ARPE-19 cells stably expressing empty vector or MYC. Expression levels of each gene was normalized to the levels of 18S, and the expression levels of each genes in ARPE control samples was set to 1. (D) Rat1 fibroblasts wild type (WT), *myc-/-* stably expressing empty vector or MYC were extracted with NP40 lysing buffer, and the total cell lysates were subjected to Western blotting with the indicated antibodies. (E) ARPE-19 cells stably expressing empty vector or MYC were extracted with NP40 lysing buffer, and the total cell lysates were subjected to Western blotting with the indicated antibodies. (F) DLD1 cells were transfected with control, MYC, or AHR siRNAs for 3 days before total protein extraction and Western blot assays. * p<0.05, ** p<0.01, *** p<0.001.
