# Supplementary material for: Induction of LEF1 by MYC activates the WNT pathway and maintains cell proliferation
Source: Cell Commun Signal. 2019 Oct 17;17:129. doi: 10.1186/s12964-019-0444-1 (PMC6798382; doi:10.1186/s12964-019-0444-1)
Supplement: Supplementary file 2 — Additional file 2: Figure S2. (A) Comparison of the levels of the indicated genes in cancer tissues and adjacent normal tissues from TCGA database. Blue: downregulated (< 0.7), grey: no change (0.7–1.3), and red: upregulated (> 1.3). n = 41 pairs. (B) Kaplan–Meier curves comparing survival of patients with colon adenocarcinoma (COAD) divided into high and low expression levels of MYC-driven WNT signaling genes. Comparison between 30% highest and 30% lowest expression were generated using ONCLnc (http://www.oncolnc.org/). [file 12964_2019_444_MOESM2_ESM.docx]

Additional file 2: **Figure S2.** (A) Comparison of the levels of the indicated genes in cancer tissues and adjacent normal tissues from TCGA database. Blue: downregulated (<0.7), grey: no change (0.7-1.3), and red: upregulated (>1.3). n= 41 pairs. (B) Kaplan–Meier curves comparing survival of patients with colon adenocarcinoma (COAD) divided into high and low expression levels of MYC-driven WNT signaling genes. Comparison between 30% highest and 30 % lowest expression were generated using ONCLnc (<http://www.oncolnc.org/>).
