# Supplementary material for: Induction of LEF1 by MYC activates the WNT pathway and maintains cell proliferation
Source: Cell Commun Signal. 2019 Oct 17;17:129. doi: 10.1186/s12964-019-0444-1 (PMC6798382; doi:10.1186/s12964-019-0444-1)
Supplement: Supplementary file 3 — Additional file 3: Figure S3. (A) Gene levels of MYC-driven WNT signaling genes were compared between cancer tissues and normal tissues, and binary heatmaps were drawn to show the changes. Samples were sorted with p-value, and changes with p-value< 0.05 were considered statistically significant. (B) Kaplan–Meier curves comparing survival of various cancer types correlated with LEF1. Comparisons between 30% highest and 30% lowest expression were generated using ONCLnc (http://www.oncolnc.org/). [file 12964_2019_444_MOESM3_ESM.docx]

Additional file 3: **Figure S3.** (A) Gene levels of MYC-driven WNT signaling genes were compared between cancer tissues and normal tissues, and binary heatmaps were drawn to show the changes. Samples were sorted with p-value, and changes with p-value<0.05 were considered statistically significant. (B) Kaplan–Meier curves comparing survival of various cancer types correlated with LEF1. Comparisons between 30% highest and 30% lowest expression were generated using ONCLnc (<http://www.oncolnc.org/>).
