# Supplementary material for: Induction of LEF1 by MYC activates the WNT pathway and maintains cell proliferation
Source: Cell Commun Signal. 2019 Oct 17;17:129. doi: 10.1186/s12964-019-0444-1 (PMC6798382; doi:10.1186/s12964-019-0444-1)
Supplement: Supplementary file 4 — Additional file 4: Figure S4. (A) Scheme of colon cancer progression. (B) Genetic background of colonic cells used in this study. Information of cancer cell was obtained from Colorectal Cancer Atlas (http://colonatlas.org/). (C) DLD1 cells were transfected with either control or MYC siRNAs, and the relative RNA levels were measured by using RT-qPCR. (D) ChIP-seq data from the ENCODE database show that MYC and MAX binds to the LEF1 promoter in K562 and MCF10A cells. Cluster Scores (out of 1000) are shown in parentheses. (E) Rat fibroblast expressing inducible MYC expression (Tet-On) was cultured in the presence of 1 μg/ml doxycycline (Dox) for 48 h. Incubation with 25 μg/ml cycloheximide (CHX) for three hours was performed prior to RNA isolation and RT-qPCR analysis. Protein synthesis inhibition reduced LEF1 mRNA both in control and MYC expression cells, but to a less extent in MYC-expressing cells. [file 12964_2019_444_MOESM4_ESM.docx]

Additional file 4: **Figure S4.** (A) Scheme of colon cancer progression. (B) Genetic background of colonic cells used in this study. Information of cancer cell was obtained from Colorectal Cancer Atlas (http://colonatlas.org/). (C) DLD1 cells were transfected with either control or MYC siRNAs, and the relative RNA levels were measured by using RT-qPCR. (D) ChIP-seq data from the ENCODE database show that MYC and MAX binds to the LEF1 promoter in K562 and MCF10A cells. Cluster Scores (out of 1000) are shown in parentheses. (E) Rat fibroblast expressing inducible MYC expression (Tet-On) was cultured in the presence of 1 µg/ml doxycycline (Dox) for 48 hours. Incubation with 25 µg/ml cycloheximide (CHX) for three hours was performed prior to RNA isolation and RT-qPCR analysis. Protein synthesis inhibition reduced LEF1 mRNA both in control and MYC expression cells, but to a less extent in MYC-expressing cells.
