# Supplementary material for: Induction of LEF1 by MYC activates the WNT pathway and maintains cell proliferation
Source: Cell Commun Signal. 2019 Oct 17;17:129. doi: 10.1186/s12964-019-0444-1 (PMC6798382; doi:10.1186/s12964-019-0444-1)
Supplement: Supplementary file 5 — Additional file 5: Figure S5. (A) ARPE-19 cells with or without MYC overexpression were fractionated into cytosolic and nuclear fractions, and the samples were subjected to Western blotting with the indicated antibodies. (B) ARPE-19 cells with or without MYC overexpression were transfected with either control or AHR siRNA, and then fractionated for Western blotting with the indicated antibodies. [file 12964_2019_444_MOESM5_ESM.docx]

Additional file 5: **Figure S5.** (A) ARPE-19 cells with or without MYC overexpression were fractionated into cytosolic and nuclear fractions, and the samples were subjected to Western blotting with the indicated antibodies. (B) ARPE-19 cells with or without MYC overexpression were transfected with either control or AHR siRNA, and then fractionated for Western blotting with the indicated antibodies.
