# Supplementary material for: Induction of LEF1 by MYC activates the WNT pathway and maintains cell proliferation
Source: Cell Commun Signal. 2019 Oct 17;17:129. doi: 10.1186/s12964-019-0444-1 (PMC6798382; doi:10.1186/s12964-019-0444-1)
Supplement: Supplementary file 6 — Additional file 6: Figure S6. (A) RKO and Huh7 cells were transfected with control or LEF1 siRNAs for 3 days and then Western blotted. (B) 5000 ARPE-19 cells with or without MYC overexpression were plated in 12-well plates and incubated with different concentrations of ICG-001 in triplicate for 7 days. Cells were then stained with crystal violet, and the relative viabilities were qualified with ImageJ. (C) RT-qPCR for LEF1 in APRE cells infected with empty vector transfected with LEF1 siRNA. [file 12964_2019_444_MOESM6_ESM.docx]

Additional file 6: **Figure S6.** (A) RKO and Huh7 cells were transfected with control or LEF1 siRNAs for 3 days and then Western blotted. (B) 5,000 ARPE-19 cells with or without MYC overexpression were plated in 12-well plates and incubated with different concentrations of ICG-001 in triplicate for 7 days. Cells were then stained with crystal violet, and the relative viabilities were qualified with ImageJ. (C) RT-qPCR for LEF1 in APRE cells infected with empty vector transfected with LEF1 siRNA.
