# Supplementary material for: Induction of LEF1 by MYC activates the WNT pathway and maintains cell proliferation
Source: Cell Commun Signal. 2019 Oct 17;17:129. doi: 10.1186/s12964-019-0444-1 (PMC6798382; doi:10.1186/s12964-019-0444-1)
Supplement: Supplementary file 7 — Additional file 7: Figure S7. (A) Diagram of the metabolomics experiment showing Rat1 myc−/− fibroblasts with human MYC expression were transfected with either control or LEF1 siRNAs in triplicate. Cells were extracted with methanol 2 days after transfection and processed for LC/MS metabolomics. (B) Parallel dishes from the same experiment were exacted with NP40 lysis buffer and subjected to Western blotting. (C) Principal component analysis (PCA) plot was generated by SIMCA 13.0.3 to show the sample clusters, and t [1] and t [2] are variances of the samples. [file 12964_2019_444_MOESM7_ESM.docx]

Additional file 7: **Figure S7.** (A) Diagram of the metabolomics experiment showing Rat1 *myc-/-* fibroblasts with human MYC expression were transfected with either control or LEF1 siRNAs in triplicate. Cells were extracted with methanol 2 days after transfection and processed for LC/MS metabolomics. (B) Parallel dishes from the same experiment were exacted with NP40 lysis buffer and subjected to Western blotting. (C) Principal component analysis (PCA) plot was generated by SIMCA 13.0.3 to show the sample clusters, and t[1] and t[2] are variances of the samples.
