# Supplementary material for: Induction of LEF1 by MYC activates the WNT pathway and maintains cell proliferation
Source: Cell Commun Signal. 2019 Oct 17;17:129. doi: 10.1186/s12964-019-0444-1 (PMC6798382; doi:10.1186/s12964-019-0444-1)
Supplement: Supplementary file 8 — Additional file 8: Figure S8. (A) Heatmap comparing the metabolic profile of myc−/− cell reconstituted with MYC and transfected with either control siRNA or siRNA for LEF1. (B) Metabolites and metabolic pathways altered by LEF1 silencing. (C) Diagram of β-oxidation with metabolites affected by LEF1 knockdown in MYC-expressing cells and their corresponding enzymes. [file 12964_2019_444_MOESM8_ESM.docx]

Additional file 8: **Figure S8.** (A) Heatmap comparing the metabolic profile of *myc-/-* cell reconstituted with MYC and transfected with either control siRNA or siRNA for LEF1. (B) Metabolites and metabolic pathways altered by LEF1 silencing. (C) Diagram of β-oxidation with metabolites affected by LEF1 knockdown in MYC-expressing cells and their corresponding enzymes.
