# Supplementary material for: Induction of LEF1 by MYC activates the WNT pathway and maintains cell proliferation
Source: Cell Commun Signal. 2019 Oct 17;17:129. doi: 10.1186/s12964-019-0444-1 (PMC6798382; doi:10.1186/s12964-019-0444-1)
Supplement: Supplementary file 9 — Additional file 9: Table S1. List of primers. [file 12964_2019_444_MOESM9_ESM.docx]

Table S1: List of primers

| **Gene name** | **Left Primer** | **Right Primer** |
| --- | --- | --- |
| Rat Myc | GTGCTGCATGAAGAGACACC | TCAATTTCTTCCTCATCATCTTGT |
| Rat Lef1 | TGGTAAACGAGTCCGAAATCA | TGTGTTTGTCCGACCACCT |
| Rat Tcf3 | AAGGTTCCGCCTGGTCTT | GGCGTCCCTGCTGTAGTTAT |
| Rat Dkk1 | CGGGAATTACTGCAAAAACG | CAATGATGCCTTCCTCGATT |
| Rat Dvl1 | GGCGGCATCTACATTGGA | TGTTCTCAAAGTTGACATCATTCAC |
| Rat Lrp6 | CATGATACGAAAGGCACAAGAA | TCTGATTTGGAACCGAGCTT |
| Rat Fzd1 | GCTCTTCGTCTATCTGTTCATCG | GAAGAGCGACACGAAACCA |
| Rat Fzd2 | GTGTCAGTGGCCTACATTGC | CCTCAGAGAAGCGCTCGTT |
| Human MYC | CACCAGCAGCGACTCTGA | GATCCAGACTCTGACCTTTTGC |
| Human LEF1 | AAACAGGAACATCCCCACAC | TCAGAGGCTTCTTAATGTGAGGT |
| Human TCF3 | CGCAGTTCGGAGGTTCAG | AGGAGGAGCTGCTCTGGTC |
| Human DVL1 | GCACCAGCTCCTCCTCACTA | TGTCACTCTTCACCGTCAGC |
| Human DVL2 | GGGTTGGGGATTATCTGAGG | CTCGCAGGAGAGACAGCAC |
| Human LRP5 | AGCTGTGAATGTGGCCAAG | CGTGGGGTGTGAAGAAGC |
| Human LRP6 | GGCACTTACTTCCCTGCAAT | TGTAATGTGATCGCTCTGTGG |
| Human FOXQ1 | GCGGACTTTGCACTTTGAA | TTTAAGGCACGTTTGATGGA |
| 18S | ACCGCAGCTAGGAATAATGGA | GCCTCAGTTCCGAAAACCA |
| LEF1 EBox-1 | ATGATGAAGTCAAGCCACTGC | GCTTATAACCCACCGAAAGGC |
| LEF1 EBox-2 | CCTCCAGCGGGCAGC | TCCCCACTGCTTCTCCTCC |
| LEF1 EBox-3 | AGGAGAAGCAGTGGGGAGG | CGTCCTGGTTCCTCGGC |
| LEF1 -2.6Kb | ACTGGAGGTTGGCTGTTGTA | GCAATTGTTTTCACGGTCGG |
